# Randomized, double-blind, controlled trial of human anti-LIGHT monoclonal antibody in COVID-19 acute respiratory distress syndrome

**DOI:** 10.1172/JCI153173

**Published:** 2022-02-01

**Authors:** David S. Perlin, Garry A. Neil, Colleen Anderson, Inbal Zafir-Lavie, Shane Raines, Carl F. Ware, H. Jeffrey Wilkins

**Affiliations:** 1 Hackensack Meridian Health Center for Discovery and Innovation, Nutley, New Jersey, USA.; 2 Avalo Therapeutics, Wayne, Pennsylvania, USA.; 3 Laboratory of Molecular Immunology, Sanford Burnham Prebys Medical Discovery Institute, La Jolla, California, USA.

**Keywords:** COVID-19, Clinical Trials, Adaptive immunity, Cytokines, Respiration

## Abstract

**BACKGROUND:**

Severe coronavirus disease 2019 (COVID-19) is associated with a dysregulated immune response, which can result in cytokine-release syndrome and acute respiratory distress syndrome (ARDS). Patients with COVID-19–associated ARDS have elevated free serum levels of the cytokine lymphotoxin-like inducible protein that competes with glycoprotein D for herpesvirus entry on T cells (LIGHT; also known as *TNFSF14*). Such patients may benefit from LIGHT-neutralization therapy.

**METHODS:**

This randomized, double-blind, multicenter, proof-of-concept trial enrolled adults hospitalized with COVID-19–associated pneumonia and mild to moderate ARDS. Patients received standard of care plus a single dose of a human LIGHT–neutralizing antibody (CERC-002) or placebo. The primary endpoint was the proportion of patients receiving CERC-002 who remained alive and free of respiratory failure through day 28. Safety was assessed via adverse event monitoring.

**RESULTS:**

For most of the 83 enrolled patients, standard of care included systemic corticosteroids (88.0%) or remdesivir (57.8%). A higher proportion of patients remained alive and free of respiratory failure through day 28 after receiving CERC-002 (83.9%) versus placebo (64.5%; *P* = 0.044), including in patients 60 years of age or older (76.5% vs. 47.1%, respectively; *P* = 0.042). Mortality rates were 7.7% (CERC-002) and 14.3% (placebo) on day 28 and 10.8% and 22.5%, respectively, on day 60. Treatment-emergent adverse events were less frequent with CERC-002 than placebo.

**CONCLUSION:**

For patients with COVID-19–associated ARDS, adding CERC-002 to standard-of-care treatment reduces LIGHT levels and might reduce the risk of respiratory failure and death.

**TRIAL REGISTRATION:**

ClinicalTrials.gov NCT04412057.

**FUNDING:**

Avalo Therapeutics.

## Introduction

In the time since March 2020, when coronavirus disease 2019 (COVID-19) became a global pandemic (1, 2), millions of people have been infected with severe acute respiratory syndrome–related coronavirus 2 (SARS-CoV-2) (3). Most infected individuals are asymptomatic or have only mild to moderate symptoms. However, some patients — particularly elderly individuals and those with concurrent health conditions — are susceptible to a severe disease course involving immune cell hyperactivation and increased levels of circulating cytokines (cytokine release syndrome [CRS]; refs. 4, 5) that can lead to acute respiratory distress syndrome (ARDS) and other life-threatening symptoms (6, 7). CRS is thought to be major cause of morbidity and mortality in COVID-19 (6, 8, 9).

Considerable progress has been made in controlling the epidemic with the introduction of vaccines and other novel therapies (10–12), yet the appearance of SARS-CoV-2 variants such as B.1.1.7 (13, 14) and 501.V2 (15) appear to be associated with increased infectivity and transmission rates among humans (16). Concerns have arisen that existing vaccines and therapies may be less effective against these variants than against wild-type SARS-CoV-2 (17, 18). An urgent medical need remains for drugs that can mitigate illness for patients at risk of a severe disease course that includes CRS and ARDS.

Cytokine neutralization has been proposed as a therapeutic strategy for CRS (19). For example, elevated levels of the cytokine IL-6 are common in CRS (4, 5). For patients with COVID-19, sarilumab and tocilizumab have been studied for their efficacy in blocking the IL-6 receptor and neutralizing IL-6, respectively, but their benefit may be limited to the most severely ill patients who require organ support (20–22). Therapies that target IFN-γ (e.g., emapalumab), TNF (infliximab), IL-1β (anakinra), NF-κB (glucocorticoids), JAK-STAT3 (JAK inhibitors), and mTOR (sirolimus, rapamycin) are all being investigated for their potential use in patients with CRS (4, 19, 23, 24).

The TNF-related cytokine lymphotoxin-like inducible protein that competes with glycoprotein D for herpesvirus entry on T cells (LIGHT; also known as *TNFSF14*) functions as an activator of both innate and adaptive immune responses. LIGHT belongs to a network of cytokines and receptors that create a self-regulating host defense system and has a key role in the communication system that controls the immune response (25). More specifically, LIGHT mediates immune activation and tissue damage (26–30). In a recent study, we found that the levels of free (active) LIGHT were elevated in serum of patients with COVID-19 (31). The degree to which LIGHT was elevated correlated with disease severity. Our results were independently confirmed by 3 separate groups (32–34). The accumulated evidence suggests LIGHT might be a viable therapeutic target for patients with COVID-19. We therefore conducted a randomized, double-blind, placebo-controlled, multicenter, phase II, proof-of-concept trial to assess the efficacy and safety of CERC-002 (AVTX-002), a human LIGHT–neutralizing antibody, in the treatment of patients with COVID-19–related CRS and ARDS.

## Results

### Study population.

**Figure 1:**
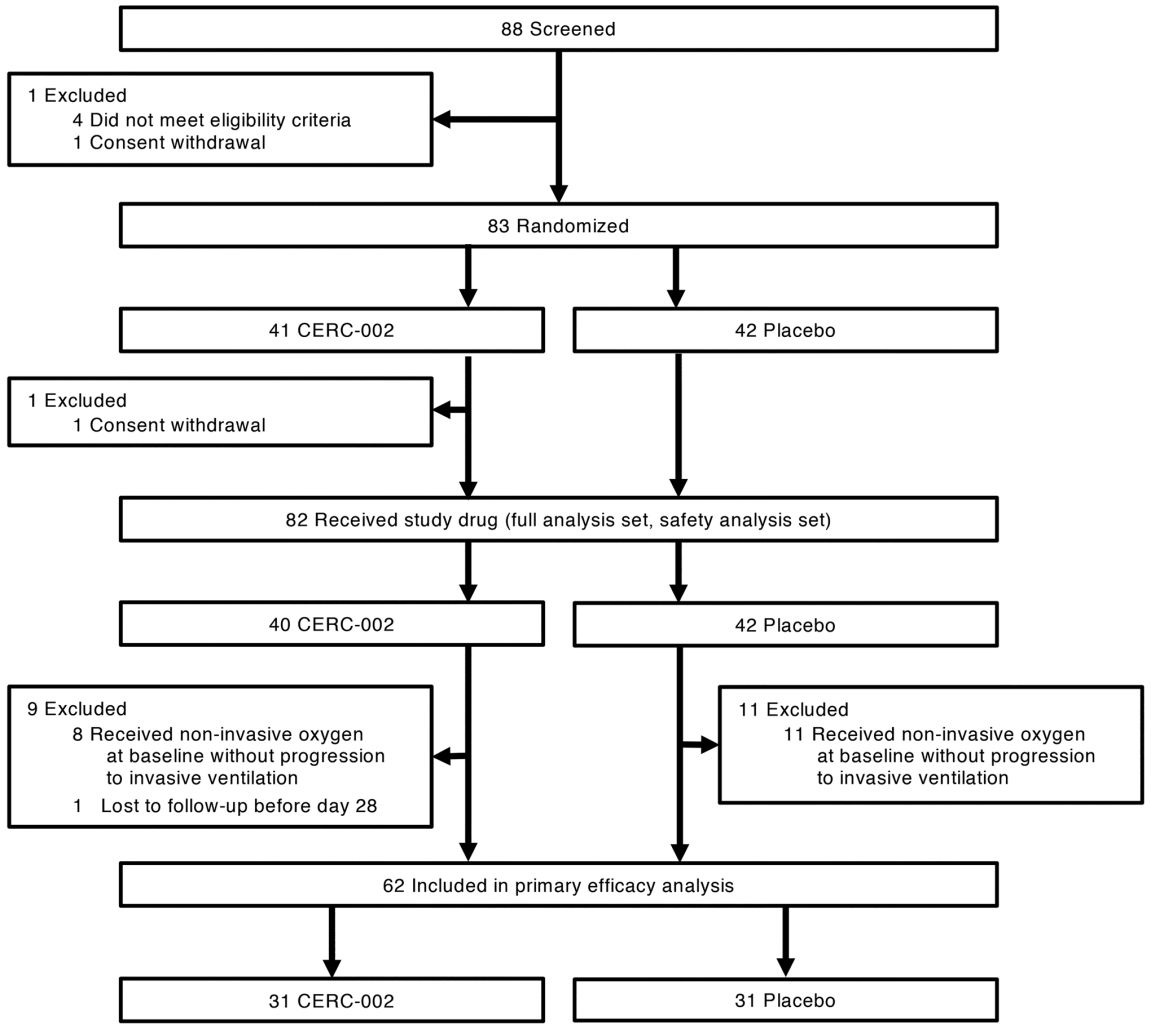
Enrollment and randomization.

The study was conducted at 11 sites across the United States between July 17, 2020 (first patient screened) and January 19, 2021 (last follow-up assessment). Eighty-three patients were randomized to receive CERC-002 (*n =* 41) or placebo (*n =* 42) (Figure 1). One patient randomized to CERC-002 was discharged before receiving the study drug. The 82 patients who received study drug were included in the full analysis and safety analysis sets, which were used for the efficacy and safety analyses, respectively. Of these 82 patients, 19 received noninvasive ventilation prior to study drug administration and were excluded from the primary efficacy analysis, as specified a priori. One patient was successfully discharged before day 28 but was lost to follow-up after discharge and did not complete the day 28 or day 60 follow-up calls. The primary endpoint was therefore analyzed using the 62 patients (*n =* 31 per treatment group) who did not experience a respiratory failure event before study drug administration or a progression to invasive ventilation after treatment.

Patient characteristics and demographics are summarized in Table 1. Among all randomized patients, the mean age was 58.7 years (50.6% ≥60 years), 31.3% were female, 81.9% were White, and the mean ± SD BMI was 33.3 ± 7.6 kg/m^2^. Most patients were receiving systemic corticosteroids (88.0%) or remdesivir (57.8%) at baseline.

### Primary outcome.

**Figure 2:**
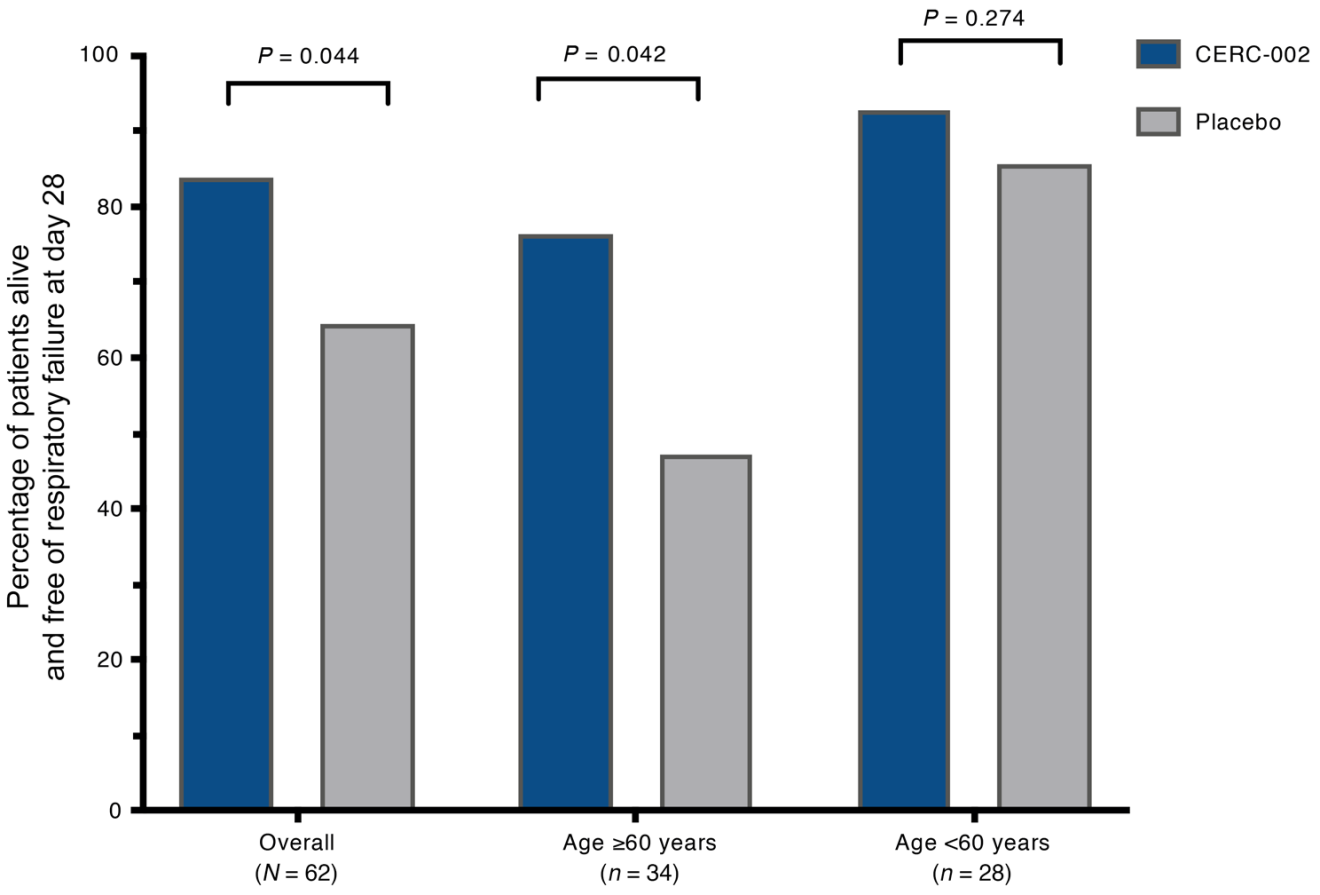
Efficacy of CERC-002 in COVID-19 patients. Percentage of patients alive and free of respiratory failure through 28 days after treatment is presented. Analysis was performed for overall (*n =* 62) patients, and separately for subgroups of patients under the age of 60 (*n =* 34) and age 60 or above (*n =* 28). One-sided *P* values were calculated using the Wald χ^2^ test.

In the primary efficacy analysis, a significantly higher percentage of patients who received CERC-002 compared with placebo remained alive and free of respiratory failure on day 28 (83.9% [26/31] vs. 64.5% [20/31]; OR [90% CI], 2.86 [1.04–7.88]; *P* = 0.044) (Figure 2). In a prespecified analysis according to patient age, the benefit with CERC-002 over placebo was maintained for patients at least 60 years old (76.5% [13/17] vs. 47.1% [8/17]; OR [90% CI], 3.66 [1.06–12.56]; *P* = 0.042). No statistically significant treatment difference was observed for patients under 60 years (92.9% [13/14] vs. 85.7% [12/14]; OR [90% CI], 2.17 [0.26–18.04]; *P* = 0.274). Among the 55 patients who were receiving concomitant corticosteroids, 82.8% (24/29; CERC-002) and 65.4% (17/26; placebo) were alive and free of respiratory failure on day 28 (OR [90% CI], 2.54 [0.88–7.30]; *P* = 0.073). Seven patients were not receiving concomitant corticosteroids during the study; of these, 100% (2/2) and 60.0% (3/5), respectively, met the primary endpoint.

### Secondary outcomes.

**Figure 3:**
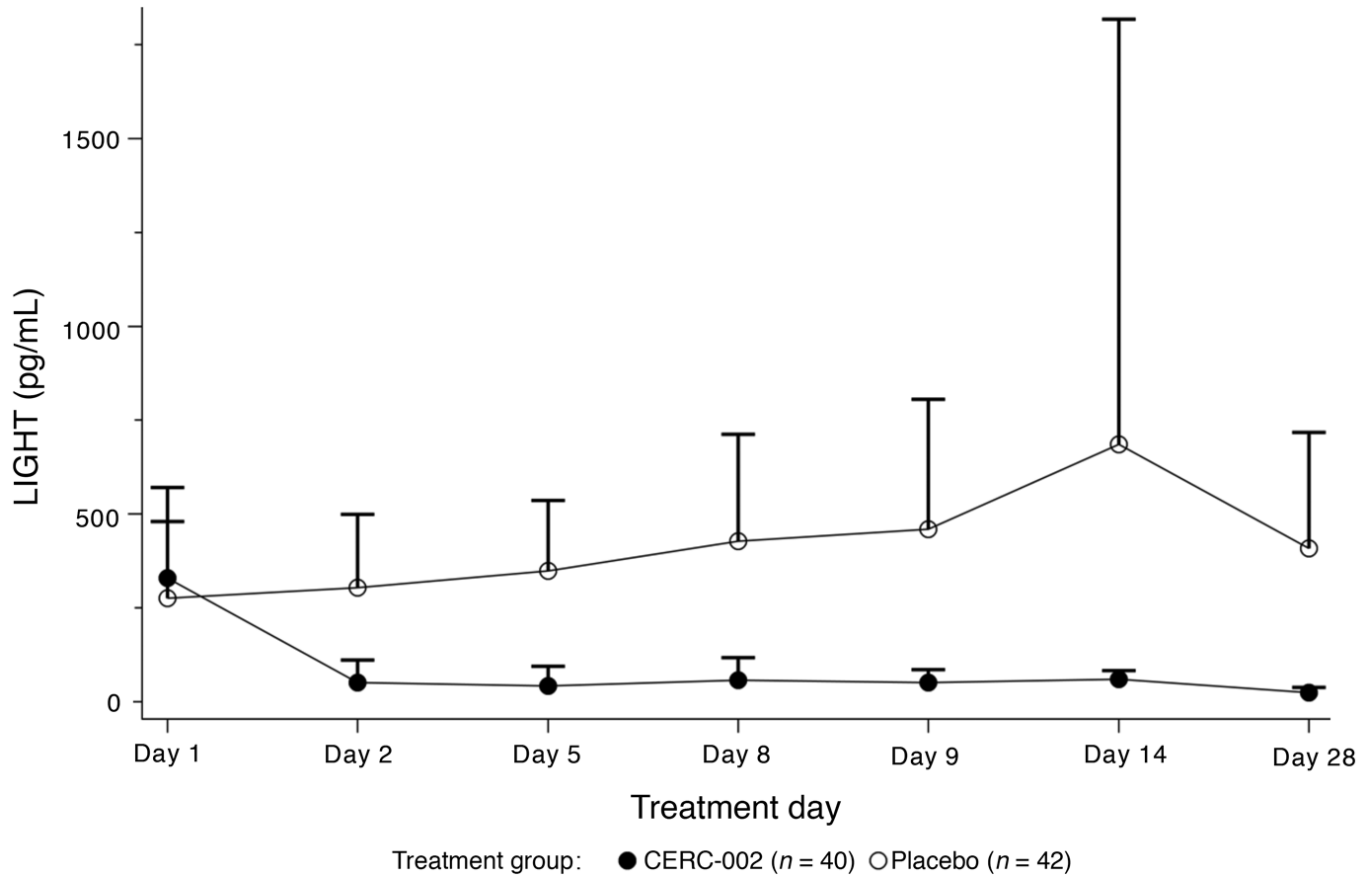
Serum free-LIGHT levels (pg/mL) over treatment period. Mean free-LIGHT levels were comparable at baseline across treatment groups. Data represent mean + SD.

In the full analysis set, 90.0% (36/40) of patients who received CERC-002 and 81.0% (34/42) who received placebo were free of invasive ventilation through day 28 (OR [90% CI], 2.12 [0.72–6.24]; *P* = 0.127). No statistically significant differences were observed between treatments for subgroup analyses according to age (<60 years vs. ≥60 years) or concomitant corticosteroid use. Similarly, among patients for whom data were available at the 28-day and 60-day follow-up points, 92.3% (36/39) and 85.7% (36/42) patients, respectively, remained alive on day 28 (OR [90% CI], 2.00 [0.59–6.82]; *P* = 0.176), with no treatment differences observed in subgroup analyses. These results suggest a possible reduction in mortality rate on day 28 of approximately 50% for patients who received CERC-002 (7.7% [3/39]) compared with placebo (14.3% [6/42]), an effect that was maintained at the 60-day safety follow-up (10.8% [4/37] vs. 22.5% [9/40], respectively). Free-LIGHT levels declined significantly (80%) and rapidly in the CERC-002 group, whereas small increases were observed in the placebo group (Figure 3).

### Safety and tolerability.

CERC-002 was generally well tolerated in the critically ill study population. Adverse events (AEs) were reported for 45.1% (37/82) of patients overall, and the types and frequencies of AEs were similar between treatment groups (Table 2). No evidence was observed for increased rates of infection or immunosuppression-related AEs with CERC-002 compared with placebo. AEs were considered possibly or probably related to study drug for 20.0% (8/40) of patients who received CERC-002 and 14.3% (6/42) who received placebo. Serious AEs, which occurred for 20.0% (8/40) and 28.6% (12/42) of patients, respectively, were considered by the investigator to be treatment related for 2 patients in the CERC-002 group (myocardial infarction and acute respiratory failure, *n =* 1 each) and 6 patients in the placebo group (respiratory failure, *n =* 2; pulseless electrical activity, hypotension, bradycardia, ventricular fibrillation, *n =* 1 each). However, the Data and Safety Monitoring Board and the sponsor judged the serious AEs to be symptoms of COVID-19 and unrelated to study drug. AEs led to discontinuation of study drug for 5.0% (2/40) patients in the CERC-002 group (respiratory failure and acute myocardial infarction, *n =* 1 each) and 11.9% (5/42) in the placebo group (respiratory failure, septic shock, cardiac arrest, and acute respiratory failure, *n =* 2 each; pulseless electrical activity, ventricular fibrillation, and hypotension, *n =* 1 each).

## Discussion

To our knowledge, this is the first clinical study to investigate the use of a LIGHT-neutralizing therapy as a treatment for hospitalized patients with COVID-19–related pneumonia with ARDS. In this study, CERC-002 was associated with a substantial reduction in respiratory failure, mortality, and serum LIGHT levels. CERC-002 provided incremental improvement over placebo for patients who were already receiving standard-of-care treatment, 88.0% of whom were also receiving systemic corticosteroids and 57.8% of whom were receiving remdesivir. CERC-002 was well tolerated and was not associated with an increased frequency of opportunistic infection or treatment-related serious AEs.

Immune hyperactivity including cytokine storm is often observed in patients with COVID-19. Because SARS-CoV-2 primarily infects the lung, patients with COVID-19–related CRS often exhibit pulmonary symptoms such as acute lung injury and ARDS, and many succumb to the disease (35). Systemic corticosteroids have been shown to be effective in treating COVID-19–related ARDS (36–40), reducing mortality rates by about 20% to 35% (36, 37). Based on such evidence, steroids became an integral part of standard-of-care treatment for patients with COVID-19–related ARDS. However, corticosteroid use is associated with considerable risk, particularly for patients who are elderly, diabetic, or immunocompromised. In addition, minimal evidence is available for using corticosteroids in patients with a milder disease course (i.e., without ARDS and/or not requiring mechanical ventilation; ref. 41). Results from the current study suggested that CERC-002 may provide benefit for patients regardless of whether concomitant corticosteroids are used, without increasing the risk of immunocompromise. Treatment benefit appears to be strongest for patients who are at least 60 years old.

Treatment approaches are therefore evolving as our understanding of the COVID-19 immune profile deepens. Evidence is accumulating to support the use of cytokine-neutralizing agents in patients with COVID-19. Several cytokine-neutralizing strategies have been assessed for the treatment of COVID-19–related ARDS and CRS. These include antagonists of IL-6 and IL-6 receptor (e.g., sarilumab, tocilizumab; refs. 20–22), IL-1β and IL-1β receptor (e.g., anakinra; ref. 42), GM-CSF (e.g., namilumab, sargramostim; ref. 43), and VEGF (e.g., bevacizumab; ref. 44). Though initial results are promising, some of these agents are still in clinical development, and additional controlled studies are required to demonstrate conclusive efficacy in patients with COVID-19. Sarilumab and tocilizumab are associated with reducing the number of days that patients require respiratory or cardiovascular organ support, but no consistent benefit has been observed for mortality rates (20–22). In our study, we identified elevated IL-6 levels that were not influenced by corticosteroid therapy or CERC-002. In addition, CERC-002 provided clinical benefit regardless of whether patients were receiving corticosteroids. These data suggest LIGHT neutralization might influence the disease course independently of IL-6. As CERC-002 does not appear to acutely influence IL-6 levels, it is possible that a combination of LIGHT- and IL-6–neutralizing therapies might increase the clinical benefit for patients.

The LIGHT cytokine is well known for its multifaceted role in immune-activating pathways and immune system regulation. Among its varied roles, LIGHT is involved in costimulating T cells (45), orchestrating fibrosis (26), and controlling autoimmunity (46). We recently reported that elevated free-LIGHT levels in the serum of patients with COVID-19–related ARDS were correlated with disease course severity (31). Moreover, expression patterns of the LIGHT receptor herpes virus entry mediator (HVEM; also known as *TNFRSF14*) in myeloid cells and in tissue barrier epithelial cells suggests that excessive LIGHT levels might cause an accumulation of neutrophils, macrophages, and T cells that promote tissue destruction (47). This possibility is supported by evidence suggesting that LIGHT has a role in pulmonary fibrosis (26). Activated T cells, macrophages, and neutrophils compose a primary source of LIGHT (48), and these cell types have been reported to infiltrate the lungs during SARS-CoV-2 infection (49). Moreover, LIGHT has a role in pulmonary inflammation that is driven by viral infection, and its levels correlate with disease severity (27, 50). Together, this evidence supports the involvement of LIGHT in COVID-19–related ARDS and CRS, and provides a rationale for using LIGHT as a therapeutic target in this context.

Several study limitations are worthy of note. The study was designed to use broad yet relevant eligibility criteria that allowed for rapid patient screening. This included the provision that patients might have received high-flow oxygen or positive-pressure oxygen prior to randomization. The primary endpoint, the proportion of patients alive and free of respiratory failure on day 28, was set according to the advice of the US FDA. Given these factors, some overlap was expected between the eligibility criteria and the primary endpoint. To address the potential overlap, an amendment was instituted before the study began that elevated the endpoint to include patients who were alive on day 28 without mechanical ventilation. The primary efficacy analysis was therefore restricted to patients who either did not experience respiratory failure before study drug administration or who required an elevation in their ventilation support. As a result, 20 patients who experienced respiratory failure before study drug administration (and/or did not require elevated support) were excluded from the primary analysis.

In addition, this phase II study was intended to provide proof of concept for CERC-002 in treating patients with COVID-19–related ARDS. Given the association between increased cytokine release and a more severe disease course, it was considered unlikely that inhibition of LIGHT with CERC-002 would negatively affect patients. Therefore, to increase statistical power in this small study, it was decided to use a 1-sided χ^2^ test to analyze the primary endpoint. The authors acknowledge the slight possibility that CERC-002 might have contributed to a more severe disease course, but that the data statistically significantly favored a treatment benefit over placebo suggests this was not the case.

In conclusion, this phase II proof-of-concept study provides initial evidence that using a specific monoclonal antibody (CERC-002) to neutralize the LIGHT cytokine might provide therapeutic benefit, including reducing mortality rates, for patients with COVID-19–related ARDS and CRS. Future studies in larger populations are needed to verify these findings.

## Methods

### Study design.

In this randomized, double-blind, placebo-controlled, multicenter, phase II trial (ClinicalTrials.gov number NCT04412057), patients were randomized 1:1 to receive a single subcutaneous injection of 16 mg/kg CERC-002 (maximum 1200 mg) or matching placebo (volume-matched saline) in addition to standard-of-care (SOC) treatment. SOC treatment, which may have included off-label use of other drugs, devices, or interventions to treat COVID-19, was continued throughout the study.

Randomization was done via Prism eSource (PRA Health Sciences) using a permuted block randomization algorithm and a block size of 2. The Prism system assigned random numbers, which were used for treatment allocation. All patients, investigators, and study personnel were blinded to treatment assignment until after the database lock, with the exception of individuals (e.g., pharmacists, individuals from the contract research organization) who required access to the randomized treatment assignment in order to fulfill their role in the study conduct and data analysis.

### Eligibility criteria.

Hospitalized adults (≥18 years old) were enrolled in the study if they had a diagnosed SARS-CoV-2 infection through an approved testing method and clinical evidence of pneumonia with acute lung injury, defined as diffuse bilateral radiographic infiltrates with a partial pressure of arterial oxygen/percentage of inspired oxygen ratio (PaO_2_/FiO_2_) above 100 and below 300 (i.e., mild to moderate ARDS). If data on oxygen saturation were available, the value at rest in ambient air must have been below 93%. Patients were permitted to receive high-flow oxygen or positive-pressure oxygen prior to randomization.

Patients were excluded from the study if they were intubated with mechanical ventilation, currently taking immunomodulators or antirejection medications, had received an immunomodulating biologic drug within 60 days of baseline, were in septic shock defined as persistent hypotension requiring vasopressors to maintain mean arterial pressure of 65 mmHg or higher and a serum lactate level above 2 mmol/L (18 mg/dL) despite adequate volume resuscitation, or had received any live attenuated vaccine, such as varicella-zoster, oral polio, or rubella, within 3 months prior to the baseline visit. Pregnant or lactating females were also excluded.

### Study objectives and endpoints.

The primary study objective was to evaluate the effect of CERC-002 compared with placebo, in addition to SOC, in preventing severe ARDS in adults with COVID-19–associated pneumonia and acute lung injury. Per the direction of the US FDA, the primary efficacy endpoint was the proportion of subjects who were alive and free of respiratory failure through day 28. As specified a priori, the primary endpoint was evaluated among patients who did not receive high-flow oxygen or positive-pressure oxygen prior to randomization, although patients receiving noninvasive oxygen support were not excluded from the study or other secondary endpoints. Respiratory failure in the primary endpoint was considered to have occurred if patients had a new-onset requirement for at least one of the following: endotracheal intubation and mechanical ventilation or extracorporeal membrane oxygenation.

Secondary objectives were to evaluate the safety, tolerability, and effects on mortality of CERC-002 compared with placebo. Secondary objectives were evaluated in all randomized patients who received treatment and had a baseline and at least 1 post-baseline efficacy assessment. Secondary efficacy endpoints, evaluated through day 28, included the proportion of patients who were free of invasive ventilation up to the day 28/early termination visit, and the proportion of patients who survived to the day 28/early termination and day 60 visits. Pharmacodynamics were assessed by evaluating changes from baseline through day 28 in serum free-LIGHT levels. Safety and tolerability were assessed through day 60 by monitoring for AEs and changes in clinical laboratory values, physical examination findings, and ECG results.

### Measurement of serum cytokine levels.

Free-LIGHT levels were measured on day 1 before study drug administration, and on days 2, 5, 8, 9, 14, and 28. Free-LIGHT assays were performed by Myriad RBM Inc. using the Quanterix fully automated HD-1 Analyzer and single molecule array (Simoa) technology, as described previously (51). All incubations were done at room temperature in the Simoa HD-1 analyzer. Capture antibody–conjugated paramagnetic beads were incubated with standards, samples, or controls and biotinylated detection antibodies. The beads were then washed and incubated with streptavidin–β-galactosidase. After the final wash, beads were loaded into the Simoa Disc with enzyme substrate (resorufin β-galactopyranoside). Fluorescence signals were compared to the standard curve, and the quantity of free LIGHT was determined for each sample. The lower and upper limits of detection for free LIGHT were determined to be 0.8 and 4000 pg/mL, respectively. IL-6 serum levels were measured as a part of the Myriad RBM Human InflammationMAP assay, using a Luminex platform, according to the manufacturer’s protocol.

### Statistics.

The study was planned to randomize a total of 82 patients at a 1:1 ratio to receive CERC-002 or placebo in addition to SOC. The sample size was selected to provide greater than 80% power to detect a 25% difference in the proportion of patients alive and free of respiratory failure on day 28, using a 1-sided significance level of 0.05. The calculation assumed that the proportions of patients alive and free of respiratory failure would be 60% in the placebo group and 85% in the CERC-002 group.

Baseline data were analyzed using the randomized analysis set, defined as all patients who were randomized in the study. The full analysis set included all randomized patients who received treatment and had a baseline and at least 1 post-baseline efficacy assessment. The primary endpoint was analyzed using a subset of the full analysis set that included patients who did not require noninvasive ventilation prior to administration of study drug or those that required noninvasive ventilation but had progression to invasive ventilation. Safety data were analyzed using the safety analysis set, defined as all randomized patients who received study treatment.

All efficacy and safety variables were summarized using descriptive statistics, such as mean, median, and range (for continuous data) or percentages of patients (for categorical data). Summaries of changes from baseline included only patients who had both a baseline value and a corresponding value at the time point of interest. For analysis of the primary efficacy endpoint, a logistic regression model was used that included a fixed effect for treatment group and provided a point estimate (i.e., OR), 90% CI, and 1-sided *P* value using the Wald χ^2^ test.

### Study approval.

Before the study was initiated and investigational product was released to each site, the study protocol, informed consent forms, and any related subject information or study materials were approved by the Advarra IRB (for 10 study sites) and the IRB at Roper–St. Francis Healthcare (1 site). All patients or a legally authorized representative provided written informed consent and assent (as applicable) to participate before undergoing screening for study eligibility.

## Author contributions

HJW and GAN conceived the study design and guided the study. CA, and SR were responsible for data extraction and verification. SR analyzed the data and constructed figures. GAN, IZL, SR, DSP, and HJW wrote this manuscript and organized tables and figures. CFW contributed to data analysis and interpretation of the results, and editing the manuscript. All authors reviewed and edited the manuscript, and approved the final version.

## Supplementary Material

Trial reporting checklists

ICMJE disclosure forms

## Figures and Tables

**Table 2:**
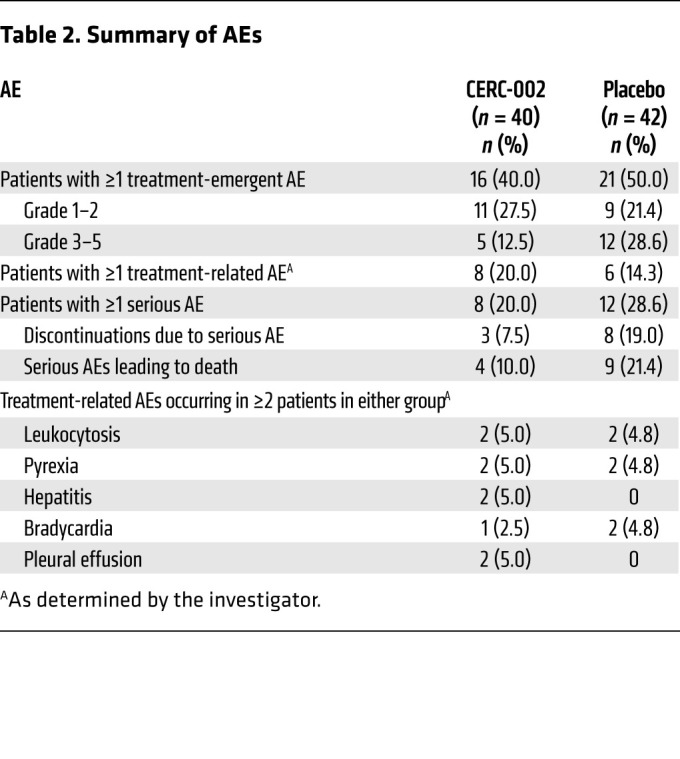
Summary of AEs

**Table 1:**
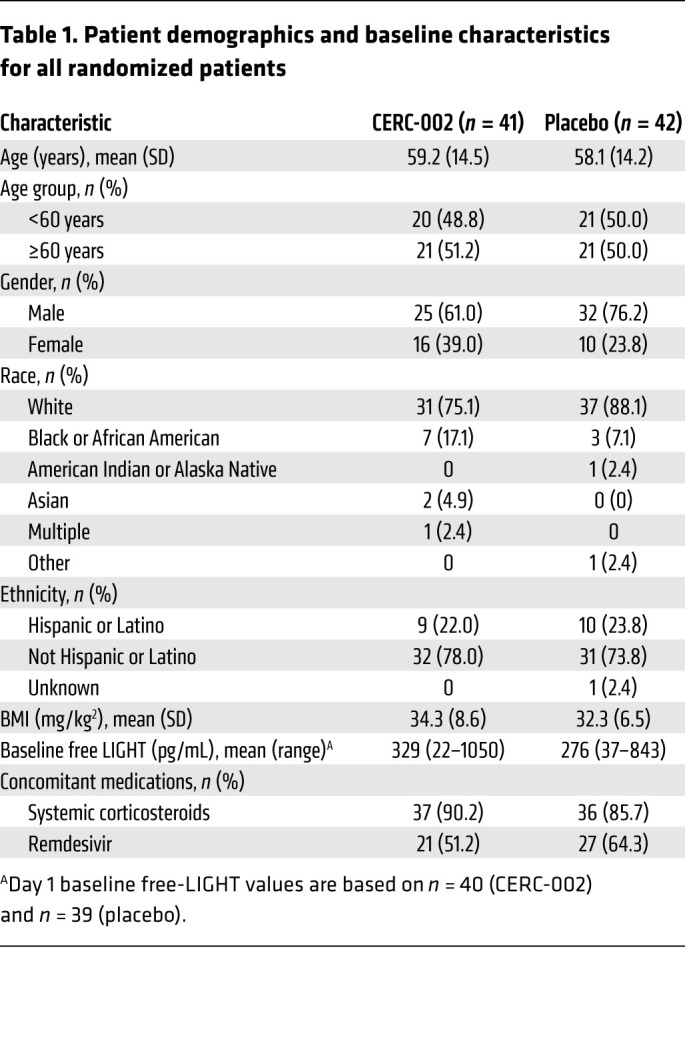
Patient demographics and baseline characteristics for all randomized patients

## Version 1 12/06/2021

In-Press Preview

## Version 2 02/01/2022

Electronic publication

## References

[1] Parr J (2020). Pneumonia in China: lack of information raises concerns among Hong Kong health workers. BMJ.

[2] Hui DS (2020). The continuing 2019-nCoV epidemic threat of novel coronaviruses to global health — The latest 2019 novel coronavirus outbreak in Wuhan, China. Int J Infect Dis.

[3] https://covid19.who.int.

[4] Fajgenbaum DC, June CH (2020). Cytokine storm. N Engl J Med.

[5] Wang W (2020). Definition and risks of cytokine release syndrome in 11 critically ill COVID-19 patients with pneumonia: analysis of disease characteristics. J Infect Dis.

[6] Huang C (2020). Clinical features of patients infected with 2019 novel coronavirus in Wuhan, China. Lancet.

[7] Chen N (2020). Epidemiological and clinical characteristics of 99 cases of 2019 novel coronavirus pneumonia in Wuhan, China: a descriptive study. Lancet.

[8] Challen R (2021). Risk of mortality in patients infected with SARS-CoV-2 variant of concern 202012/1: matched cohort study. BMJ.

[9] Hojyo S (2020). How COVID-19 induces cytokine storm with high mortality. Inflamm Regen.

[10] Sanders JM (2020). Pharmacologic treatments for coronavirus disease 2019 (COVID-19): a review. JAMA.

[11] Izda V (2021). COVID-19: a review of therapeutic strategies and vaccine candidates. Clin Immunol.

[12] https://www.who.int/publications/m/item/draft-landscape-of-covid-19-candidate-vaccines.

[13] Wise J (2020). Covid-19: New coronavirus variant is identified in UK. BMJ.

[14] Frampton D (2021). Genomic characteristics and clinical effect of the emergent SARS-CoV-2 B.1.1.7 lineage in London, UK: a whole-genome sequencing and hospital-based cohort study. Lancet Infect Dis.

[15] Wibmer CK (2021). SARS-CoV-2 501Y.V2 escapes neutralization by South African COVID-19 donor plasma. Nat Med.

[16] https://www.who.int/csr/don/31-december-2020-sars-cov2-variants/en/.

[17] Koyama T (2020). Emergence of drift variants that may affect COVID-19 vaccine development and antibody treatment. Pathogens.

[18] Wang P (2021). Antibody resistance of SARS-CoV-2 variants B.1.351 and B.1.1.7. Nature.

[19] Shimabukuro-Vornhagen A (2018). Cytokine release syndrome. J Immunother Cancer.

[20] Remap-Cap Investigators (2021). Interleukin-6 receptor antagonists in critically ill patients with Covid-19. N Engl J Med.

[21] Salama C (2021). Tocilizumab in patients hospitalized with Covid-19 pneumonia. N Engl J Med.

[22] Campochiaro C (2020). Efficacy and safety of tocilizumab in severe COVID-19 patients: a single-centre retrospective cohort study. Eur J Intern Med.

[23] Bischof E (2021). The potential of rapalogs to enhance resilience against SARS-CoV-2 infection and reduce the severity of COVID-19. Lancet Healthy Longev.

[24] Magro G (2020). COVID-19: Review on latest available drugs and therapies against SARS-CoV-2. Coagulation and inflammation cross-talking. Virus Res.

[25] Ward-Kavanagh LK (2016). The TNF receptor superfamily in co-stimulating and co-inhibitory responses. Immunity.

[26] Herro R, Croft M (2016). The control of tissue fibrosis by the inflammatory molecule LIGHT (TNF superfamily member 14). Pharmacol Res.

[27] Doherty TA (2011). The tumor necrosis factor family member LIGHT is a target for asthmatic airway remodeling. Nat Med.

[28] Desai P (2018). The TNF superfamily molecule LIGHT promotes the generation of circulating and lung-resident memory CD8 T cells following an acute respiratory virus infection. J Immunol.

[29] Wang J (2005). The critical role of LIGHT in promoting intestinal inflammation and Crohn’s disease. J Immunol.

[30] del Rio ML (2013). LIGHT/HVEM/LTbetaR interaction as a target for the modulation of the allogeneic immune response in transplantation. Am J Transplant.

[31] Perlin DS (2020). Levels of the TNF-related cytokine LIGHT increase in hospitalized COVID-19 patients with cytokine release syndrome and ARDS. mSphere.

[32] Arunachalam PS (2020). Systems biological assessment of immunity to mild versus severe COVID-19 infection in humans. Science.

[33] Haljasmagi L (2020). Longitudinal proteomic profiling reveals increased early inflammation and sustained apoptosis proteins in severe COVID-19. Sci Rep.

[35] Henderson LA (2020). On the alert for cytokine storm: immunopathology in COVID-19. Arthritis Rheumatol.

[36] Recovery Collaborative Group (2021). Dexamethasone in hospitalized patients with Covid-19. N Engl J Med.

[37] WHO Rapid Evidence Appraisal for COVID-19 Therapies Working Group (2020). Association between administration of systemic corticosteroids and mortality among critically ill patients with COVID-19: A meta-analysis. JAMA.

[38] Angus DC (2020). Effect of hydrocortisone on mortality and organ support in patients with severe COVID-19: the REMAP-CAP COVID-19 Corticosteroid Domain Randomized Clinical Trial. JAMA.

[39] Dequin PF (2020). Effect of hydrocortisone on 21-day mortality or respiratory support among critically ill patients with COVID-19: a randomized clinical trial. JAMA.

[40] Tomazini BM (2020). Effect of dexamethasone on days alive and ventilator-free in patients with moderate or severe acute respiratory distress syndrome and COVID-19: the CoDEX randomized clinical trial. JAMA.

[41] Shuto H (2020). A systematic review of corticosteroid treatment for noncritically ill patients with COVID-19. Sci Rep.

[42] Cavalli G, Dagna L (2021). The right place for IL-1 inhibition in COVID-19. Lancet Respir Med.

[43] Lang FM (2020). GM-CSF-based treatments in COVID-19: reconciling opposing therapeutic approaches. Nat Rev Immunol.

[44] Pang J (2021). Efficacy and tolerability of bevacizumab in patients with severe Covid-19. Nat Commun.

[45] Mortarini R (2005). Constitutive expression and costimulatory function of LIGHT/TNFSF14 on human melanoma cells and melanoma-derived microvesicles. Cancer Res.

[46] Sonar S, Lal G (2015). Role of tumor necrosis factor superfamily in neuroinflammation and autoimmunity. Front Immunol.

[47] Schwarz BT (2007). LIGHT signals directly to intestinal epithelia to cause barrier dysfunction via cytoskeletal and endocytic mechanisms. Gastroenterology.

[48] Edwards JP (2006). Biochemical and functional characterization of three activated macrophage populations. J Leukoc Biol.

[49] Channappanavar R, Perlman S (2017). Pathogenic human coronavirus infections: causes and consequences of cytokine storm and immunopathology. Semin Immunopathol.

[50] Xu W (2019). Transcriptome sequencing identifies novel immune response genes highly related to the severity of human adenovirus type 55 infection. Front Microbiol.

[51] Rodrigues KB (2020). Innate immune stimulation of whole blood reveals IFN-1 hyper-responsiveness in type 1 diabetes. Diabetologia.

