# Supplementary material for: Randomized, double-blind, controlled trial of human anti-LIGHT monoclonal antibody in COVID-19 acute respiratory distress syndrome
Source: J Clin Invest. 2022 Feb 1;132(3):e153173. doi: 10.1172/JCI153173 (PMC8803341; doi:10.1172/JCI153173)
Supplement: ICMJE disclosure forms [file jci-132-153173-s126.pdf]

ICMJE DISCLOSURE FORM

Date: October 5, 2021
Your Name: Colleen Anderson
Manuscript Title: Randomized, double-blind, controlled trial of human anti-LIGHT monoclonal antibody in COVID-19 acute respiratory distress syndrome
Manuscript number (if known): 153173-JCI-CMED-1

In the interest of transparency, we ask you to disclose all relationships/activities/interests listed below that are related to the content of your manuscript. "Related" means any relation with for-profit or not-for-profit third parties whose interests may be affected by the content of the manuscript. Disclosure represents a commitment to transparency and does not necessarily indicate a bias. If you are in doubt about whether to list a relationship/activity/interest, it is preferable that you do so.

The following questions apply to the author's relationships/activities/interests as they relate to the current manuscript only.

The author's relationships/activities/interests should be defined broadly. For example, if your manuscript pertains to the epidemiology of hypertension, you should declare all relationships with manufacturers of antihypertensive medication, even if that medication is not mentioned in the manuscript.

In item #1 below, report all support for the work reported in this manuscript without time limit. For all other items, the time frame for disclosure is the past 36 months.

		**Name all entities with whom you have this relationship or indicate none (add rows as needed)**	**Specifications/Comments (e.g., if payments were made to you or to your institution)**
		**Time frame: Since the initial planning of the work**	
1	All support for the present manuscript (e.g., funding, provision of study materials, medical writing, article processing charges, etc.) **No time limit for this item.**	_X___ None	
		**Time frame: past 36 months**	
2	Grants or contracts from any entity (if not indicated in item #1 above).	_X___ None	
3	Royalties or licenses	_X___ None	

4	Consulting fees	_X___ None	
5	Payment or honoraria for lectures, presentations, speakers bureaus, manuscript writing or educational events	_X___ None	
6	Payment for expert testimony	_X___ None	
7	Support for attending meetings and/or travel	_X___ None	
8	Patents planned, issued or pending	_X___ None	
9	Participation on a Data Safety Monitoring Board or Advisory Board	_X___ None	
10	Leadership or fiduciary role in other board, society, committee or advocacy group, paid or unpaid	_X___ None	
11	Stock or stock options	____ None	
		Avalo Therapeutics (formerly Cerecor, Inc.)	Hold stock in Avalo Therapeutics, which funded the study
12	Receipt of equipment, materials, drugs, medical writing, gifts or other services	_X___ None	
13	Other financial or non-financial interests	_____ None	
		Avalo Therapeutics (formerly Cerecor, Inc.)	Employee of Avalo Therapeutics, which funded the study

Please place an "X" next to the following statement to indicate your agreement:

_X__ I certify that I have answered every question and have not altered the wording of any of the questions on this form.

ICMJE DISCLOSURE FORM

Date: October 5, 2021
Your Name: Carl F. Ware
Manuscript Title: Randomized, double-blind, controlled trial of human anti-LIGHT monoclonal antibody in COVID-19 acute respiratory distress syndrome
Manuscript number (if known): 153173-JCI-CMED-1

In the interest of transparency, we ask you to disclose all relationships/activities/interests listed below that are related to the content of your manuscript. "Related" means any relation with for-profit or not-for-profit third parties whose interests may be affected by the content of the manuscript. Disclosure represents a commitment to transparency and does not necessarily indicate a bias. If you are in doubt about whether to list a relationship/activity/interest, it is preferable that you do so.

The following questions apply to the author's relationships/activities/interests as they relate to the current manuscript only.

The author's relationships/activities/interests should be defined broadly. For example, if your manuscript pertains to the epidemiology of hypertension, you should declare all relationships with manufacturers of antihypertensive medication, even if that medication is not mentioned in the manuscript.

In item #1 below, report all support for the work reported in this manuscript without time limit. For all other items, the time frame for disclosure is the past 36 months.

		**Name all entities with whom you have this relationship or indicate none (add rows as needed)**	**Specifications/Comments (e.g., if payments were made to you or to your institution)**
**Time frame: Since the initial planning of the work**			
1	All support for the present manuscript (e.g., funding, provision of study materials, medical writing, article processing charges, etc.) **No time limit for this item.**	_X___ None	
**Time frame: past 36 months**			
2	Grants or contracts from any entity (if not indicated in item #1 above).	_X___ None	
3	Royalties or licenses	La Jolla Institute	Royalty payment

4	Consulting fees	Avalo Therapeutics	Consulting fee
5	Payment or honoraria for lectures, presentations, speakers bureaus, manuscript writing or educational events	_X___ None	
6	Payment for expert testimony	_X___ None	
7	Support for attending meetings and/or travel	_X___ None	
8	Patents planned, issued or pending	La Jolla Institute	Inventor and issued patent related to anti-LIGHT
9	Participation on a Data Safety Monitoring Board or Advisory Board	_X___ None	
10	Leadership or fiduciary role in other board, society, committee or advocacy group, paid or unpaid	_X___ None	
11	Stock or stock options	Avalo Therapeutics	Stock options
12	Receipt of equipment, materials, drugs, medical writing, gifts or other services	_X___ None	
13	Other financial or non-financial interests	_X___ None	

Please place an "X" next to the following statement to indicate your agreement:

_X__ I certify that I have answered every question and have not altered the wording of any of the questions on this form.

ICMJE DISCLOSURE FORM

Date: October 5, 2021
Your Name: David S. Perlin
Manuscript Title: Randomized, double-blind, controlled trial of human anti-LIGHT monoclonal antibody in COVID-19 acute respiratory distress syndrome
Manuscript number (if known): 153173-JCI-CMED-1

In the interest of transparency, we ask you to disclose all relationships/activities/interests listed below that are related to the content of your manuscript. "Related" means any relation with for-profit or not-for-profit third parties whose interests may be affected by the content of the manuscript. Disclosure represents a commitment to transparency and does not necessarily indicate a bias. If you are in doubt about whether to list a relationship/activity/interest, it is preferable that you do so.

The following questions apply to the author's relationships/activities/interests as they relate to the <u>current manuscript only</u>.

The author's relationships/activities/interests should be <u>defined broadly</u>. For example, if your manuscript pertains to the epidemiology of hypertension, you should declare all relationships with manufacturers of antihypertensive medication, even if that medication is not mentioned in the manuscript.

In item #1 below, report all support for the work reported in this manuscript without time limit. For all other items, the time frame for disclosure is the past 36 months.

		**Name all entities with whom you have this relationship or indicate none (add rows as needed)**	**Specifications/Comments (e.g., if payments were made to you or to your institution)**
		**Time frame: Since the initial planning of the work**	
1	All support for the present manuscript (e.g., funding, provision of study materials, medical writing, article processing charges, etc.) **No time limit for this item.**	_X___ None	
		**Time frame: past 36 months**	
2	Grants or contracts from any entity (if not indicated in item #1 above).	_X___ None	
3	Royalties or licenses	_X___ None	

4	Consulting fees	_X___ None	
5	Payment or honoraria for lectures, presentations, speakers bureaus, manuscript writing or educational events	_X___ None	
6	Payment for expert testimony	_X___ None	
7	Support for attending meetings and/or travel	_X___ None	
8	Patents planned, issued or pending	_X___ None	
9	Participation on a Data Safety Monitoring Board or Advisory Board	_X___ None	
10	Leadership or fiduciary role in other board, society, committee or advocacy group, paid or unpaid	_X___ None	
11	Stock or stock options	_X___ None	
12	Receipt of equipment, materials, drugs, medical writing, gifts or other services	_X___ None	
13	Other financial or non-financial interests	_X___ None	

Please place an "X" next to the following statement to indicate your agreement:

_X__ I certify that I have answered every question and have not altered the wording of any of the questions on this form.

ICMJE DISCLOSURE FORM

Date: October 5, 2021
Your Name: Garry A. Neil
Manuscript Title: Randomized, double-blind, controlled trial of human anti-LIGHT monoclonal antibody in COVID-19 acute respiratory distress syndrome
Manuscript number (if known): 153173-JCI-CMED-1

In the interest of transparency, we ask you to disclose all relationships/activities/interests listed below that are related to the content of your manuscript. "Related" means any relation with for-profit or not-for-profit third parties whose interests may be affected by the content of the manuscript. Disclosure represents a commitment to transparency and does not necessarily indicate a bias. If you are in doubt about whether to list a relationship/activity/interest, it is preferable that you do so.

The following questions apply to the author's relationships/activities/interests as they relate to the current manuscript only.

The author's relationships/activities/interests should be defined broadly. For example, if your manuscript pertains to the epidemiology of hypertension, you should declare all relationships with manufacturers of antihypertensive medication, even if that medication is not mentioned in the manuscript.

In item #1 below, report all support for the work reported in this manuscript without time limit. For all other items, the time frame for disclosure is the past 36 months.

		**Name all entities with whom you have this relationship or indicate none (add rows as needed)**	**Specifications/Comments (e.g., if payments were made to you or to your institution)**
		**Time frame: Since the initial planning of the work**	
1	All support for the present manuscript (e.g., funding, provision of study materials, medical writing, article processing charges, etc.) **No time limit for this item.**	_X___ None	
		**Time frame: past 36 months**	
2	Grants or contracts from any entity (if not indicated in item #1 above).	_X___ None	
3	Royalties or licenses	_X___ None	

4	Consulting fees	_X___ None	
5	Payment or honoraria for lectures, presentations, speakers bureaus, manuscript writing or educational events	_X___ None	
6	Payment for expert testimony	_X___ None	
7	Support for attending meetings and/or travel	_X___ None	
8	Patents planned, issued or pending	_X___ None	
9	Participation on a Data Safety Monitoring Board or Advisory Board	_X___ None	
10	Leadership or fiduciary role in other board, society, committee or advocacy group, paid or unpaid	_X___ None	
11	Stock or stock options	____ None	
		Avalo Therapeutics (formerly Cerecor, Inc.)	Hold stock in Avalo Therapeutics, which funded the study
12	Receipt of equipment, materials, drugs, medical writing, gifts or other services	_X___ None	
13	Other financial or non-financial interests	_____ None	
		Avalo Therapeutics (formerly Cerecor, Inc.)	Employee of Avalo Therapeutics, which funded the study

Please place an "X" next to the following statement to indicate your agreement:

_X__ I certify that I have answered every question and have not altered the wording of any of the questions on this form.

ICMJE DISCLOSURE FORM

Date: October 5, 2021
Your Name: H. Jeffrey Wilkins
Manuscript Title: Randomized, double-blind, controlled trial of human anti-LIGHT monoclonal antibody in COVID-19 acute respiratory distress syndrome
Manuscript number (if known): 153173-JCI-CMED-1

In the interest of transparency, we ask you to disclose all relationships/activities/interests listed below that are related to the content of your manuscript. "Related" means any relation with for-profit or not-for-profit third parties whose interests may be affected by the content of the manuscript. Disclosure represents a commitment to transparency and does not necessarily indicate a bias. If you are in doubt about whether to list a relationship/activity/interest, it is preferable that you do so.

The following questions apply to the author's relationships/activities/interests as they relate to the current manuscript only.

The author's relationships/activities/interests should be defined broadly. For example, if your manuscript pertains to the epidemiology of hypertension, you should declare all relationships with manufacturers of antihypertensive medication, even if that medication is not mentioned in the manuscript.

In item #1 below, report all support for the work reported in this manuscript without time limit. For all other items, the time frame for disclosure is the past 36 months.

		**Name all entities with whom you have this relationship or indicate none (add rows as needed)**	**Specifications/Comments (e.g., if payments were made to you or to your institution)**
		**Time frame: Since the initial planning of the work**	
1	All support for the present manuscript (e.g., funding, provision of study materials, medical writing, article processing charges, etc.) **No time limit for this item.**	_X___ None	
		**Time frame: past 36 months**	
2	Grants or contracts from any entity (if not indicated in item #1 above).	_X___ None	
3	Royalties or licenses	_X___ None	

4	Consulting fees	_X___ None	
5	Payment or honoraria for lectures, presentations, speakers bureaus, manuscript writing or educational events	_X___ None	
6	Payment for expert testimony	_X___ None	
7	Support for attending meetings and/or travel	_X___ None	
8	Patents planned, issued or pending	_X___ None	
9	Participation on a Data Safety Monitoring Board or Advisory Board	_X___ None	
10	Leadership or fiduciary role in other board, society, committee or advocacy group, paid or unpaid	_X___ None	
11	Stock or stock options	____ None	
		Avalo Therapeutics (formerly Cerecor, Inc.)	Hold stock in Avalo Therapeutics, which funded the study
12	Receipt of equipment, materials, drugs, medical writing, gifts or other services	_X___ None	
13	Other financial or non-financial interests	_____ None	
		Avalo Therapeutics (formerly Cerecor, Inc.)	Employee of Avalo Therapeutics, which funded the study

Please place an "X" next to the following statement to indicate your agreement:

_X__ I certify that I have answered every question and have not altered the wording of any of the questions on this form.

ICMJE DISCLOSURE FORM

Date: October 5, 2021
Your Name: Inbal Zafir-Lavie
Manuscript Title: Randomized, double-blind, controlled trial of human anti-LIGHT monoclonal antibody in COVID-19 acute respiratory distress syndrome
Manuscript number (if known): 153173-JCI-CMED-1

In the interest of transparency, we ask you to disclose all relationships/activities/interests listed below that are related to the content of your manuscript. "Related" means any relation with for-profit or not-for-profit third parties whose interests may be affected by the content of the manuscript. Disclosure represents a commitment to transparency and does not necessarily indicate a bias. If you are in doubt about whether to list a relationship/activity/interest, it is preferable that you do so.

The following questions apply to the author's relationships/activities/interests as they relate to the current manuscript only.

The author's relationships/activities/interests should be defined broadly. For example, if your manuscript pertains to the epidemiology of hypertension, you should declare all relationships with manufacturers of antihypertensive medication, even if that medication is not mentioned in the manuscript.

In item #1 below, report all support for the work reported in this manuscript without time limit. For all other items, the time frame for disclosure is the past 36 months.

		**Name all entities with whom you have this relationship or indicate none (add rows as needed)**	**Specifications/Comments (e.g., if payments were made to you or to your institution)**
		**Time frame: Since the initial planning of the work**	
1	All support for the present manuscript (e.g., funding, provision of study materials, medical writing, article processing charges, etc.) **No time limit for this item.**	_X___ None	
		**Time frame: past 36 months**	
2	Grants or contracts from any entity (if not indicated in item #1 above).	_X___ None	
3	Royalties or licenses	_X___ None	

4	Consulting fees	_X___ None	
		Avalo Therapeutics (formerly Cerecor, Inc.)	Paid consultant to Avalo Therapeutics, which funded the study
5	Payment or honoraria for lectures, presentations, speakers bureaus, manuscript writing or educational events	_X___ None	
6	Payment for expert testimony	_X___ None	
7	Support for attending meetings and/or travel	_X___ None	
8	Patents planned, issued or pending	_X___ None	
9	Participation on a Data Safety Monitoring Board or Advisory Board	_X___ None	
10	Leadership or fiduciary role in other board, society, committee or advocacy group, paid or unpaid	_X___ None	
11	Stock or stock options	_X___ None	
12	Receipt of equipment, materials, drugs, medical writing, gifts or other services	_X___ None	
13	Other financial or non-financial interests	_X___ None	

Please place an "X" next to the following statement to indicate your agreement:

_X__ I certify that I have answered every question and have not altered the wording of any of the questions on this form.

ICMJE DISCLOSURE FORM

Date: October 5, 2021
Your Name: Shane Raines
Manuscript Title: Randomized, double-blind, controlled trial of human anti-LIGHT monoclonal antibody in COVID-19 acute respiratory distress syndrome
Manuscript number (if known): 153173-JCI-CMED-1

In the interest of transparency, we ask you to disclose all relationships/activities/interests listed below that are related to the content of your manuscript. "Related" means any relation with for-profit or not-for-profit third parties whose interests may be affected by the content of the manuscript. Disclosure represents a commitment to transparency and does not necessarily indicate a bias. If you are in doubt about whether to list a relationship/activity/interest, it is preferable that you do so.

The following questions apply to the author's relationships/activities/interests as they relate to the current manuscript only.

The author's relationships/activities/interests should be defined broadly. For example, if your manuscript pertains to the epidemiology of hypertension, you should declare all relationships with manufacturers of antihypertensive medication, even if that medication is not mentioned in the manuscript.

In item #1 below, report all support for the work reported in this manuscript without time limit. For all other items, the time frame for disclosure is the past 36 months.

		**Name all entities with whom you have this relationship or indicate none (add rows as needed)**	**Specifications/Comments (e.g., if payments were made to you or to your institution)**
**Time frame: Since the initial planning of the work**			
1	All support for the present manuscript (e.g., funding, provision of study materials, medical writing, article processing charges, etc.) **No time limit for this item.**	_X___ None	
**Time frame: past 36 months**			
2	Grants or contracts from any entity (if not indicated in item #1 above).	_X___ None	
3	Royalties or licenses	_X___ None	

4	Consulting fees	_X___ None	
		Avalo Therapeutics (formerly Cerecor, Inc.)	Paid consultant to Avalo Therapeutics, which funded the study
5	Payment or honoraria for lectures, presentations, speakers bureaus, manuscript writing or educational events	_X___ None	
6	Payment for expert testimony	_X___ None	
7	Support for attending meetings and/or travel	_X___ None	
8	Patents planned, issued or pending	_X___ None	
9	Participation on a Data Safety Monitoring Board or Advisory Board	_X___ None	
10	Leadership or fiduciary role in other board, society, committee or advocacy group, paid or unpaid	_X___ None	
11	Stock or stock options	_X___ None	
12	Receipt of equipment, materials, drugs, medical writing, gifts or other services	_X___ None	
13	Other financial or non-financial interests	_X___ None	

Please place an "X" next to the following statement to indicate your agreement:

_X__ I certify that I have answered every question and have not altered the wording of any of the questions on this form.
